# Characterizing the Action-Observation Network Through Functional Near-Infrared Spectroscopy: A Review

**DOI:** 10.3389/fnhum.2021.627983

**Published:** 2021-02-18

**Authors:** Emma E. Condy, Helga O. Miguel, John Millerhagen, Doug Harrison, Kosar Khaksari, Nathan Fox, Amir Gandjbakhche

**Affiliations:** ^1^Eunice Kennedy Shriver National Institute of Child Health and Human Development (NICHD), National Institutes of Health, Bethesda, MD, United States; ^2^Department of Human Development and Quantitative Methodology, University of Maryland, College Park, MD, United States

**Keywords:** fNIRS, action-observation, motor, mirror neuron, mu rhythm, neuroimaging

## Abstract

Functional near-infrared spectroscopy (fNIRS) is a neuroimaging technique that has undergone tremendous growth over the last decade due to methodological advantages over other measures of brain activation. The action-observation network (AON), a system of brain structures proposed to have “mirroring” abilities (e.g., active when an individual completes an action or when they observe another complete that action), has been studied in humans through neural measures such as fMRI and electroencephalogram (EEG); however, limitations of these methods are problematic for AON paradigms. For this reason, fNIRS is proposed as a solution to investigating the AON in humans. The present review article briefly summarizes previous neural findings in the AON and examines the state of AON research using fNIRS in adults. A total of 14 fNIRS articles are discussed, paying particular attention to methodological choices and considerations while summarizing the general findings to aid in developing better protocols to study the AON through fNIRS. Additionally, future directions of this work are discussed, specifically in relation to researching AON development and potential multimodal imaging applications.

## Introduction

Interactions in everyday life require us to continuously encode the actions and intentions of others. It has been suggested this ability to interpret the actions of others requires the involvement of our own motor system and is mediated by a distinct class of neuronal cells historically referred to as mirror neurons (Rizzolatti and Sinigaglia, 2016). Mirror neurons were first reported in the macaque monkey ventral premotor region (di Pellegrino et al., 1992; Rizzolatti, 2005) and inferior parietal lobe (IPL; Fogassi et al., 2005) using a single-neuron recording, while the monkey observed and executed simple actions. Their defining characteristic is neuronal firing both when individuals perform a given action and when an individual observes someone perform the same or similar action (di Pellegrino et al., 1992; Gallese et al., 1996; Fogassi et al., 2005). The discovery of mirror neurons led to new theories regarding how primates generate actions and monitor and interpret the actions of others (Rizzolatti and Sinigaglia, 2010). Moreover, it prompted the idea that perceptual and motor processes share a common neural code that facilitates the recognition and imitation of other’s actions (Fox et al., 2016), and that similar neurons may exist in the human brain.

The theoretical mirror neuron system (MNS) in humans has been associated with processes as social cognition, namely empathy (Gutsell and Inzlicht, 2010; Perry et al., 2010), the theory of mind (Pineda and Hecht, 2009), biological motion (Ulloa and Pineda, 2007), and language (Théoret and Pascual-Leone, 2002; Tamura et al., 2012; Jenson et al., 2014). Moreover, the putative role of MNS in action representation, action understanding, and imitation skills led researchers to link this system with neurodevelopment and neurodevelopmental disorders characterized by deficits in these domains, namely autism (Williams et al., 2001; Oberman et al., 2005). Despite the number of studies examining the role of the MNS in action understanding theory and cognitive processes, there is still an ongoing debate in the field regarding the existence and role of these cells or regions in the human brain (Hickok, 2009). One primary reason this question remains is a lack of adequate neuroimaging methods for measuring brain activity during the conditions necessary to investigate the MNS (e.g., motor execution) in humans (Dinstein, 2008; Fox et al., 2016).

In contrast with animal models, non-invasive neuroimaging techniques, namely functional magnetic resonance imaging (fMRI) and electroencephalogram (EEG) have largely been used to study the human mirroring system. This literature has limitations and is criticized for frequent lack of appropriate experimental designs, often due to the restrictions of the chosen neural measurements (which will be discussed further in the context of this review article). These limitations have led to criticism of the “mirror neuron” nomenclature in humans, as there is not yet convincing evidence that: (1) mirror properties are present at the neuronal level in humans (Turella et al., 2009); and (2) mirroring properties are represented in specific brain regions. Instead, the action-observation network (AON) is proposed as a more appropriate designation, framing the problem as an investigation of two separate networks (action execution and observation networks) whose overlapping structures remain to be determined. For the remainder of the present review article, we will refer to this action execution and observation system as the AON, as this terminology more accurately reflects the state of the literature which does not yet thoroughly assess the “mirroring” capacity of the network.

Although fMRI and EEG have historically been used to examine the AON in humans, they have limitations in the information they can provide. In this article, we recommend the use of functional near-infrared spectroscopy (fNIRS) as a neuroimaging technique to study the AON in humans. We start by providing a brief overview of the current state of the EEG and fMRI literature on the AON, as well as a discussion of the advantages and disadvantages of each modality. We then conduct a review of 14 fNIRS studies that have examined the AON in adults, detailing their methods and findings, as well as a discussion of their limitations and suggestions for optimizing fNIRS data collection in AON studies. Finally, we discuss the potential utility of using fNIRS in combination with other techniques and how a multi-modal imaging approach can provide a better understanding of the AON.

### The AON Through Electroencephalography (EEG)

EEG has been widely used to study the AON through the quantification of event-related desynchronization (ERD) in the 8–12 Hz frequency band at central scalp sites, which is referred to as *mu desynchronization* (Kuhlman, 1978). Mu desynchronization occurs during both observation of an action and execution of that same action (Hobson and Bishop, 2016), though the effect is stronger in execution conditions. For this reason, mu desynchronization has become a prevalent measure for assessing AON activity in humans. However, many EEG studies aimed at assessing AON activity exclude crucial elements from their paradigms, such as including only an execution or observation condition but not both (Fox et al., 2016). Furthermore, the strength of mu suppression during action observation appears sensitive to specific features of the stimuli, such as perspective (Frenkel-Toledo et al., 2013), action experience (e.g., mu suppression only occurs when the subject has experience acting (Cannon et al., 2014; Toriyama et al., 2018), and modality of the stimulus (Ruysschaert et al., 2013; Cuevas et al., 2014); e.g., live actors elicit more activation than video stimuli). For these reasons, findings regarding AON activity using EEG have been critiqued and need further investigation through more rigorous methods.

Additionally, there is an ongoing debate regarding whether mu desynchronization reflects AON activity, or specifically “mirroring” activity in the brain, or whether this can be attributed to confounding signals. Mu rhythm shares topography with the beta rhythm during motor paradigms (McFarland et al., 2000; Simon and Mukamel, 2016) and the frequency band of the alpha rhythm (Anderson and Ding, 2011), both of which contribute confounding activation that may interfere with making conclusions about AON activity. A major critique is that alpha rhythm, which is associated with attentional processes, shows widespread desynchronization during AON paradigms, and thus may not specifically relate to activity at central scalp regions (Hobson and Bishop, 2016). While studies have shown robust 8–12 Hz desynchronization at central scalp sites (thus, interpreted as mu rhythm) during both action execution and observation, desynchronization in this frequency band also appears in frontal and occipital regions during these conditions (Perry and Bentin, 2009; Marshall and Meltzoff, 2011; Debnath et al., 2019), indicating global alpha desynchrony. This lack of spatial resolution interferes with the ability to pinpoint neural structures (let alone single neurons as shown in the non-human primate MNS model) while also calling into question the construct being tapped (e.g., action-observation vs. attention). These issues can be partially addressed through high-density EEG configurations and source localization models, however, this approach incurs additional concerns with compliance during the cap application process, particularly in studies with developmental populations. Therefore, a modality with a comparatively greater spatial resolution with similar paradigm flexibility to EEG would be desirable and informative. To address the issues of confounding activity, action-observation specificity, and source localization in using mu in AON research, functional magnetic resonance imaging (fMRI) has also been used to study action-observation paradigms.

### The AON Through Functional Magnetic Resonance Imaging (fMRI)

fMRI has been utilized to localize brain regions associated with action-observation paradigms that are consistent with mu suppression activity as assessed by EEG (Song et al., 2015). Due to higher spatial resolution, fMRI is useful in detecting brain regions involved in AON that remain elusive when using EEG (Morales et al., 2019). Several review articles have revealed consistent neural correlates of the AON through fMRI (Caspers et al., 2010; Molenberghs et al., 2012; Savaki and Raos, 2019), including the ventral premotor cortex (PMC), IPL, superior temporal sulcus (STS), superior parietal lobule (SPL), and middle frontal gyrus (MFG; Koehler et al., 2012; Savaki and Raos, 2019). However, many of these studies have not included execution conditions in their paradigms. For example, a meta-analysis of AON studies found only 22 (~30%) of studies included in their meta-analysis included an action execution condition (Molenberghs et al., 2012). This is likely due to fMRI’s high sensitivity to motion artifact, making action execution conditions challenging. However, this also prevents comparisons of execution and observation conditions, limiting the ability to draw meaningful conclusions about whether these are regions pertinent to the AON. As previously noted, AON activity cannot be delineated without both an action execution and observation condition (Fox et al., 2016; Savaki and Raos, 2019), highlighting a major limitation of using fMRI to understand the AON (Field et al., 2000; Filimon et al., 2007). Additionally, even when action execution conditions are integrated into these fMRI studies of the AON, they often lack ecological validity. Examples of actions used include biting a custom-made bite bar (Filimon et al., 2007), or squeezing a ball (Jelsone-Swain et al., 2015), which might not be representative of action execution in everyday life. Overall, the confines of fMRI procedures are not ideal for adequate experimental designs for examining the AON, inciting the need for new methodologies that can improve upon these limitations.

### Using fNIRS to Study the Action-Observation Network

fNIRS is an optical imaging technique that uses light in the near-infrared range to provide measurements of changes in oxygenated hemoglobin (HbO) and deoxygenated hemoglobin (HHb; Hoshi, 2003). More specifically, the concentration of these two chromophores can be measured by using two different wavelengths of near-infrared light (e.g., 700–1,000 nm). The basic fNIRS mechanism requires a source optode, emitting light, and a detector optode, measuring the backscatter of that emitted light from the surrounding tissue, that is applied to the surface from which a measurement is taken. While this method has existed to measure these chromophores in tissue for decades, more recently it has been applied to the study of neural tissue by applying these optodes to the scalp. By placing arrays of sources and detectors across the head, blood oxygenation across cortical regions can be measured. There are some limitations to this technology, such as the depth of measurement due to the scattering properties of extracerebral tissues (e.g., skull, cerebrospinal fluid) and accurate localization of these measurements to cortical regions (Hoshi, 2003). However, the technology does offer some advantages compared to other commonly used neuroimaging techniques that make it particularly beneficial for studying the AON.

The use of fNIRS in neuroimaging has risen in recent years due to its affordability, portability, and relative tolerance to motion artifacts compared with other modalities. These considerations are particularly important in the context of the AON due to the task demands involved in AON paradigms, namely measurement of neural activation associated with motor output (Perrey, 2008; Ferrari and Quaresima, 2012). Furthermore, most AON research is conducted in the context of investigating various processes throughout development, such as social functioning. For this reason, technologies examining the AON should be capable of being effectively employed across a variety of developmental stages, including infancy and toddlerhood, to promote the feasibility of longitudinal or cross-sectional study designs. Due to the limitations regarding imaging children and motor activity using fMRI, EEG has largely been used to evaluate the AON in these populations, yielding spatially vague neural activation patterns as discussed earlier. However, fNIRS offers increased compliance in younger children and infants (Lloyd-Fox et al., 2010) and increased spatial resolution compared to EEG (Bunge and Kahn, 2009), two issues that have historically interfered with the use of neuroimaging in developmental research. Furthermore, like fMRI and EEG, fNIRS can be also be used to investigate connectivity patterns in the cortex (Anwar et al., 2016), making it a viable technology not only for examining cortical activation but also functional connectivity. Subsequently, the present review aimed to determine whether current literature using fNIRS to assess the adult AON has used consistent methods and produced coherent findings, along with outlining methodological standards for consideration when applying fNIRS to investigate the AON with other populations (e.g., infants, toddlers) in the future.

### Search Criteria

**Figure 1:**
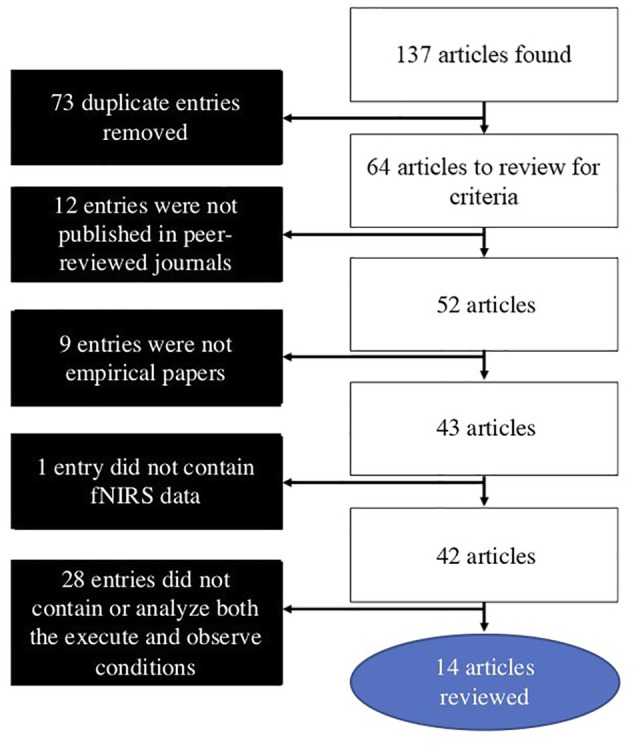
Literature search and article selection process.

The search for the present review was conducted on July 9, 2019. Searches were conducted in PubMed, Web of Science, and Scopus databases. The following combination of search terms was used: [(near-infrared spectroscopy OR fNIRS OR NIRS OR near-infrared spectroscopy) AND (mirror neuron OR action observation OR action-observation OR action/observation OR execution observation OR execution-observation OR execution/observation)]. These searches yielded 137 results, with 64 of these being novel articles. Articles were then individually reviewed using the following inclusion criteria: (1) published in a peer-reviewed journal; (2) an empirical article; (3) used fNIRS to measure hemodynamic activity in the brain; and (4) contained and analyzed both an execute and observe condition in the AON paradigm. After these criteria were applied, 14 articles remained and were included in the present review article, which offers a critical literature review article. A flowchart outlining the search and inclusion process is presented in Figure 1.

### Methodological Considerations

A summary of details regarding the methodology and findings from the articles that were included in the present review article is provided in Supplementary Table 1.

#### fNIRS Systems and Optode Placement

**Figure 2:**
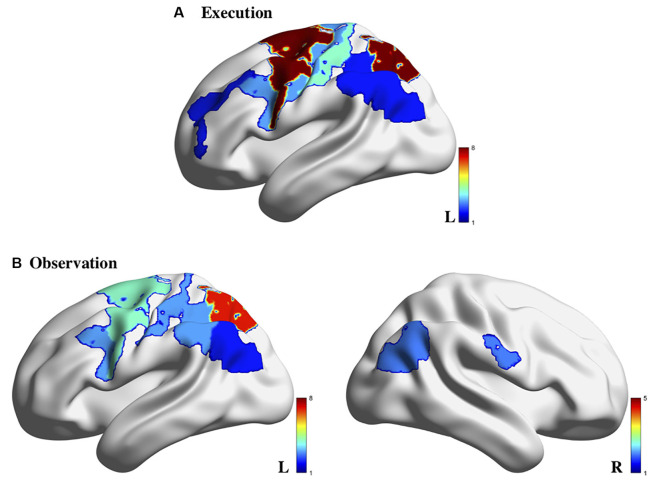
Brain activity associated with action execution **(A)** and action observation **(B)** measured using fNIRS in the 14 studies reviewed. Note: the color bar refers to the number of studies for which the brain region was indicated to be activated. Image generated using BrainNet Viewer software (Xia et al., 2013).

A variety of fNIRS systems were used across the AON studies. These systems included NIRx systems (5/14; including the NIRScout and NIRSport models), Hitachi systems (4/14; including the ETG-100 and ETG-4000 models), Shimadzu systems (4/14; including the LABNIRS and OMM-3000 models), and a custom-built system (1/14). The fNIRS systems used across these studies employed between 8 and 54 (*median* = 24) channels across various regions of the cortex. In terms of hemispheric coverage placement, there were fewer studies that utilized a bilateral probe (5/14; 35.75%): (Holper et al., 2010; Kajiume et al., 2013; Bhat et al., 2017; Crivelli et al., 2018; Zhang et al., 2019) compared to unilateral probes (9/14; 64.3%): (Shimada and Abe, 2010; Egetemeir et al., 2011; Koehler et al., 2012; Balconi et al., 2015, 2017; Balconi and Cortesi, 2016; Sun et al., 2018; Xu et al., 2019). The wide use of unilateral probes is likely due to the wide variety of regions implicated in the AON that these studies aimed to cover. Using a unilateral probe allows for a larger array of AON related cortical regions to be covered than if the limited number of fNIRS channels had been split to cover both hemispheres. While many targeted the sensorimotor cortex (SM1), other regions of interest included the IFG, IPL, PMC in only the contralateral hemisphere (see Supplementary Table 1 for a summary of ROIs covered in each study). As summarized previously, the variety of regions covered across these articles is largely due to the wide number of areas that have been implicated in the AON through other modalities such as EEG and fMRI. However, it is evident when looking at the results of the studies that used bilateral probes that lateralization differences may provide an important distinction between observing and execute conditions in AON studies (Figure 2). Multiple studies found that activation during the observation condition appeared bilaterally (Holper et al., 2010; Bhat et al., 2017) or in ipsilateral regions (Crivelli et al., 2018). For this reason, when designing an fNIRS study targeted at probing the AON, researchers should be aware that a bilateral probe may be better suited to uncovering important network characteristics. It should be noted that all the studies in the present review article only included right-handed participants, making the use of a unilateral probe methodologically acceptable; still, the exclusion of all left-handed participants, an issue across all of neuroimaging (Willems et al., 2014), limits the generalizability of these findings and should be avoided in future studies.

#### Paradigm Design

In determining which studies were eligible under the present review article, a striking number of studies were removed because they did not include both an execute and observe condition (28/42; 66.7%). These were studies that were interested in probing the AON based on our initial search criteria but did not adequately do so due to missing key conditions in their experimental paradigms, even though fNIRS is perhaps the technology best suited to assessing motor conditions (e.g., execute conditions). Future studies should be mindfully designed to ensure that at minimum a clear observe and execute condition are included in the paradigm to properly probe the AON.

The types of paradigms used across the studies varied widely, including whether objects were used (e.g., rock-paper-scissors or intransitive gestures vs. table-setting or transitive gestures) and what type of stimuli were presented. Notably, most of the articles (10/14; 71.4%) used pre-recorded video stimuli during the observation condition instead of live-action observation of an experimenter. While this approach allows greater control of stimulus presentation during the observation condition, it has the potential to minimize AON activation elicited. Previous studies have shown that mu suppression is attenuated in adults in response to video stimuli compared to live-action stimuli during AON paradigms (Järveläinen et al., 2001) as well as in infants (Cuevas et al., 2014). Similarly, a study comparing live-action vs. video stimuli using fNIRS found that activation differences between action observation and object observation condition are only elicited when live stimuli are used in both infants and adults; when video stimuli were used similar levels of activation are observed between conditions (Shimada and Hiraki, 2006). These studies may be basing their experimental design on previous research done in fMRI, even though specific design elements (e.g., use of a monitor, lack of execution condition) are not necessary for fNIRS as they are for fMRI. Due to the methodological flexibility of fNIRS, researchers implementing AON paradigms should take full advantage of its tolerance to motion artifacts during execution conditions and its ability to record during interactions with a live confederate during observation conditions, unlike fMRI. For these reasons, AON studies should aim to use live-action stimuli whenever possible to ensure that activations are not being attenuated due to the stimulus modality.

An additional consideration regarding the methods used in the reviewed articles is the timing and number of trials that were used for each condition. The trial number and trial timing standards of EEG are not appropriate for fNIRS studies largely due to the temporal differences in EEG and fNIRS; whereas the EEG response is on the order of milliseconds, the hemodynamic response takes approximately 3–5 s to begin after the stimulus and 10–15 s to recover. If an fNIRS study had the number of trials akin to an EEG paradigm and appropriate intertrial intervals were used, this would create unwieldy fNIRS paradigm lengths, increasing concerns regarding participant fatigue and attrition. Relatedly, although fNIRS measures the hemodynamic response like fMRI, the lower signal-to-noise ratio of fNIRS makes it imperative to account for this difference in paradigm design (Cui et al., 2011) through appropriate trial numbers and environmental noise control. When reviewing the paradigm details, it is apparent that there is wide variation in the design across fNIRS studies. The fNIRS studies in the present review article contained an average of 20.21 trials per condition (*median* = 8), though the most common number of trials was eight per condition with a range of 1–54 trials administered. Most of these studies used an event-related design, as opposed to a block design to evaluate the activity of the AON. The use of an event-related design allows the entire hemodynamic response function to be modeled, which allows for multiple features of the hemodynamic response to be quantified (e.g., mean amplitude, peak amplitude, slope, time to peak) and for event-related coupling of brain activity (Orihuela-Espina et al., 2010). Block designs are used but do not take advantage of the higher sampling rate afforded by fNIRS, which permits an examination of the full-time course of the hemodynamic response. This should be kept in mind when designing future AON fNIRS studies, as trial-based paradigms may provide additional information about the AON compared to block designs, allowing for specific questions regarding the timing and coupling of brain activity to events within and between the execution and observation conditions (e.g., action planning/anticipation, the start of action) to be analyzed.

### AON Findings

For the present review article, we will focus on the findings from the 14 identified studies that pertain to the observe and execute conditions. While many of the studies contained additional conditions, the parameters of these varied widely making them difficult to compare. By focusing on the core conditions relevant to the AON, we aim to better understand which cortical areas to target in future AON studies.

A few studies noted cortical activation that appeared specific to the observation condition. Establishing a pattern of activation for each condition separately allows researchers to ensure that the proper neural response is elicited during each condition, and then determine which areas may overlap across these conditions to form the AON. In the reviewed studies, observation trials yielded greater bilateral activation compared to the execution conditions (Bhat et al., 2017; Crivelli et al., 2018). In terms of execution-specific activation, activation related to the execution condition was generally stronger than the observation condition across multiple cortical areas (Holper et al., 2010; Shimada and Abe, 2010; Koehler et al., 2012; Bhat et al., 2017). However, activation during execution was more localized to motor-specific regions such as the PMC and/or primary sensory-motor cortex (Shimada and Abe, 2010; Balconi and Cortesi, 2016; Balconi et al., 2017) than other regions. Specifically, there appears to be greater activation in contralateral motor areas than in parietal areas during the execution of a motor action (Balconi et al., 2017; Bhat et al., 2017; Crivelli et al., 2018). Relatedly, the execution of action also appears to result in bilateral premotor activation, an area implicated in action planning and does not appear to be consistently active during observation conditions (Holper et al., 2010), indicating that this may not be part of the AON.

The explicit comparison of the observation and execution conditions across these studies would be where crucial regions of the AON are revealed. Many of the studies in the present review article examined which regions were significantly different than baseline within each condition and did not directly compare the level of activation between conditions. For example, one study noted that there was a main effect of condition across the region (which included the primary somatosensory and motor areas, premotor and supplementary motor areas, IFG, and DLPFC) between an execution, observation, and imagery condition; however, they did not identify which of these regions drove the main effect to determine how the conditions may have differed in activation patterns (Zhang et al., 2019). Relatedly, Xu et al. (2019) found that channels covering left IFG left PMC, and left rostral IPL were active across both the execute and observe conditions (Xu et al., 2019) but did not address whether there were significant differences in the level of activation between the conditions. Based on the figures provided in Xu et al. (2019), it appears that these regions showed greater activation during execution than observation overall. While these studies implicate a widespread network involved in the action-observation process, they fail to thoroughly characterize the subtler differences that may occur between the observe and execute conditions. One area in particular that appears to be implicated across both conditions consistently is the parietal cortex, which is activated in both observation and execution conditions compared to baseline levels (Egetemeir et al., 2011; Koehler et al., 2012; Balconi and Cortesi, 2016; Balconi et al., 2017), indicating it may be a key region in the AON (Figure 2).

These findings show that fNIRS can be used to assess activation of the AON in adults. Not only do these studies validate the use of this method, but also reveal important methodological guidelines and considerations for future AON fNIRS studies. Importantly, the present review article did not cover the developmental literature; however, this is potentially the area in which fNIRS methods are of greatest value. Study of the AON has historically been in the context of child development due to the theoretical ties to many important processes such as language acquisition (Théoret and Pascual-Leone, 2002; Le Bel et al., 2009) and social functioning (Iacoboni and Dapretto, 2006; Oberman et al., 2007). For this reason, the use of a technology such as fNIRS which allows for neural measurement without the methodological confines of fMRI will be hugely beneficial in investigating AON development and associated outcomes in infants and children. While this approach is feasible, to the authors’ knowledge no such study has been published and should be considered an important future direction for the field. This method creates an opportunity to investigate whether, we can improve upon the spatial specificity of the methods we have previously been limited by in AON research.

## Considerations and Limitations of The fNIRS AON Literature

### Probe Configuration and Placement

While fNIRS can be a promising tool for examining AON, there are several practical considerations and limitations to the technique that must be considered. To optimally leverage the spatial resolution of fNIRS to EEG, one important consideration is the application of the fNIRS optodes on the scalp. These considerations include placing and measuring the probe relative to fiducials, choosing a proper probe configuration, and ensuring adequate scalp contact. The AON articles reviewed do not adequately describe their probe placement, nor do they report probe measurements. This is problematic as it calls into question the validity of their findings as it is unclear whether they are measuring from the cortical region they claim. Future studies must be mindful both in selecting where to place probes and in measuring and reporting the location of these arrays on the scalps of individual subjects.

To draw conclusions about which regions of the brain are active using fNIRS, the probe must be placed over appropriate cortical regions across research participants. To achieve this, researchers should be conscientious when designing their probe, creating probes with cortical coverage custom to the task they are conducting, and being sure to measure its location relative to fiducials after it has been placed. This measurement can be done in multiple ways, such as digitization of the probe using a 3D digitizer (Whalen et al., 2008), or photogrammetry optode registration (Hu et al., 2020), allowing for the projection of channels onto a brain atlas (e.g., Colin27) through software such as AtlasViewer (Aasted et al., 2015) or NIRS-SPM (Ye et al., 2009), or other MRI atlas-based approaches (Wijeakumar et al., 2015). This is particularly important in AON research due to controversy in other imaging modalities, namely EEG, about the localization of AON activity. Several of the reviewed studies did not adequately measure or describe their procedures for probe placement, making it challenging to know to which cortical region activation could be attributed. As done in EEG studies, the 10–20 system is often used in fNIRS to place probes relative to the regions of interest using external landmarks. While these can provide a regional approximation (e.g., parietal lobe, frontal lobe), accurate measurement of exact optode locations relative to fiducials is critical in progressing AON research through fNIRS, as playing to the strengths of the modality, namely its improved spatial resolution.

Relatedly, appropriate source-detector distances within a probe configuration are necessary for conducting fNIRS studies, particularly when designing custom probe sets. Several studies have investigated how large a source-detector distance is needed to ensure that the cortex is being probed (Mancini et al., 1994; Strangman et al., 2002; Sato et al., 2013; Funane et al., 2014). Notably, different scalp locations often have varying extracerebral layer compositions (e.g., skull thickness), an issue that is particularly critical when covering disparate scalp regions, as would be required in an AON study. Further source-detector distances allow for deeper cortical measurement to be taken; distances that are too short risk deriving measurements from only extracerebral layers. Some groups have considered probes designed with multiple source-detector distances to study hemodynamics at different depths (Khaksari et al., 2018). Specifically, the incorporation of short distance source-detector pairs is shown to be valuable during processing to minimize signal noise (Brigadoi and Cooper, 2015). Through the probe localization options described above, source-detector distances can be derived to ensure appropriate spacing.

### Paradigm and Study Design

In reviewing the fNIRS AON literature, the variability in action observation paradigms was evident. Variation in paradigms included parameters such as length and timing, type of action (e.g., table setting, rock paper scissors), and stimulus administration (e.g., videos, live-action). While findings across these studies were generally consistent, this amount of variation introduces noise and calls the generalizability of these results into question. Furthermore, a standardized approach would allow more specific hypotheses to be generated in future studies aiming to further detail the functionality of the AON. Additionally, many of the studies in the initial search did not end up fitting the AON criteria to be included in the present review article, largely due to a lack of both an execute and observe the condition. The lack of either of these conditions makes them inconsequential to studying the AON. Optimizing the utility of these protocols in studying the AON can be achieved through careful study planning and consideration of the literature, as this is an issue that is also seen in other neuroimaging modalities targeting the AON. Future studies should ensure that both action execution and observation conditions are included and that the paradigms that are used are consistent with standards from the fNIRS and AON literature.

Additionally, the analytic design across papers was disparate and makes their comparability suspect. While this is an issue within the field of fNIRS generally (Tak and Ye, 2014), certain analytic choices that were inconsistent are unique to AON paradigms. For example, many of the studies did not conduct comparisons between the action execution and observation conditions, instead only offering the results of each condition’s activation level compared to baseline. This approach does not provide any information about whether activation is comparable between conditions, which is important considering that AON is posited to consist of sets of regions that similarly activation in both execution and observation of action. Future fNIRS research targeting the AON should be mindful of this when determining their analytic approach to ensure that their hypotheses are clear and relevant to the AON and that these hypotheses are adequately tested through their proposed analyses.

### The Implausibility of Subcortical Measurement and Relative Spatial Resolution to fMRI

The penetration depth of fNIRS is dictated by the source-detector separation, scattering properties of extracerebral and cerebral tissues, and the ANSI standard, limiting the intensity of light infiltrating the brain. As a result, one can expect to probe depths no more than 2–3 cm in the adult brain (Liu et al., 2015). For this reason, subcortical activation cannot be measured using fNIRS, unlike fMRI. This is an important consideration when deciding whether fNIRS would be a suitable approach for a research question. In the context of this review article, the fMRI research of the AON largely only implicates cortical regions, making subcortical measurement irrelevant. Further, in terms of its spatial resolution, the spatial resolution of fNIRS is dependent upon the number and density of optical sources and detectors. Though the spatial resolution of fNIRS is superior to EEG, it is inferior to fMRI, which acquires measurements on the order of millimeters. While this decreased spatial resolution is not necessarily ideal, this tradeoff is sensible in certain situations given the motion tolerance and device mobility afforded by fNIRS, particularly in a paradigm that requires a motor execution condition such as the AON. Furthermore, while the spatial resolution of fNIRS systems may be inferior to that of fMRI, the temporal resolution is higher in fNIRS, with the ability to record with a common sampling rate of 10 Hz (Pinti et al., 2020), allowing for additional investigation regarding the timing of the hemodynamic response and neural activity.

### Signal-to-Noise Ratio and Motion Artifact

One pitfall of fNIRS is its relatively weak signal-to-noise ratio (SNR) relative to fMRI (Cui et al., 2011). There are several types of artifacts that may contaminate the fNIRS signal. Instrumental noise, experimental errors, and physiological oscillations are the main sources of artifacts. Many of these can be ameliorated *a priori* through proper paradigm design and data collection procedures. In terms of paradigm design, researchers should be sure to include an adequate number of trials to characterize the hemodynamic response associated with each experimental condition. Design efficiency should be optimized to account for this required number of trials and appropriate interstimulus interval lengths. Unlike EEG, fNIRS requires longer intertrial intervals, or recovery periods, more akin to fMRI designs due to the nature of the hemodynamic response. During data collection, the scalp should be thoroughly prepped, and the probe securely fastened to avoid data contamination due to obstruction of the light source or probe displacement, respectively. Decreased detection of optical intensities, resulting in a weaker signal, can occur when hair is present in the region where a source or detector is placed (Strangman et al., 2002). Proper scalp preparation, namely parting the hair, and displacing any hair that remains under a source or detector after the probe is placed can improve this issue. Additionally, improper, or inconsistent contact with the scalp is detrimental to fNIRS signal quality. Taking care to hold probes flush to the scalp (e.g., proper probe fitting, taking care with additional probe securement), and utilizing fastening aides to keep fNIRS fibers immobile and relieve tension from the probe set are vital in avoiding artifacts from excessive motion.

## The Future of fNIRS in Studying The AON

With the state of AON imaging considered, the use of fNIRS in studying the AON holds promise for further exploring the elusive AON with increased spatial resolution compared to EEG and improved external validity and affordability compared to fMRI. While the current literature in healthy adults indicates that AON activity can be measured through fNIRS, further exploration is warranted due to the limitations and gaps in this literature noted in the present review article. Furthermore, expanding the scope of this research to include additional measurements and/or other populations to which fNIRS is well-suited would also be beneficial in elucidating the AON’s structure, function, and development with this technology. Below, we will provide several suggestions on how fNIRS can be used to better elucidate the AON.

### Ensure Probe Placement Captures all Cortical Regions Implicated in the AON

Studies using fNIRS should capture hemodynamic data from all relevant cortical regions while also taking the methodological considerations mentioned in the present review article into account. While this review article provides evidence from fNIRS for several key cortical regions of interest, no study has attained measurements across all implicated areas bilaterally. Further, differences between the experimental paradigms used in each study were reviewed to make it difficult to determine whether the active areas found in individual studies would have been seen across all studies. For these reasons, a thorough investigation of activity across the entire cortex during both action execution and observation is warranted. Many commercially available fNIRS systems are capable of whole head optode configurations, though require the funding to purchase a system with the necessary number of sources and detectors. Using such systems will provide more specific information about AON regions, including connectivity or relative timing of activations, where previous studies have been limited.

### Cross-validate fNIRS Findings From the AON With Other Neuroimaging Modalities

fNIRS could be particularly valuable in validating other measures of AON activity, namely mu desynchronization. While an fNIRS-alone study would provide more information regarding the location of the AON, integrating these measures allows for further validation of each method when studying the AON. Such a study would provide converging evidence for the utility of each technique by revealing localized cortical areas through fNIRS that correspond with mu desynchronization from EEG, the dominant method for conducting AON research that has had its validity questioned due to lack of spatial specificity. Confirming this relationship would assist in settling the debate in the literature regarding mu desynchronization and the AON. fNIRS is particularly well-suited for this task because it can monitor cortical activity in combination with electrical fields with no interference between instruments. Previous studies attempted to tackle this by finding links between EEG and fMRI signals in the context of the AON (Arnstein et al., 2011), however, such research suffers from the limitations of conducting AON research in the fMRI environment that were previously discussed, and is significantly challenged logistically when compared to combining EEG and fNIRS. Not only would combined EEG-fNIRS studies strengthen previous EEG findings in the AON, but merging the temporal and spatial resolution of both signals could more precisely delineate which brain regions are activated following the execution and observation of action. Leveraging the complementary facets of information from these simultaneous data streams would allow for more advanced analyses using multivariate approaches to fusion analysis, such as joint independent component analysis and canonical correlation analysis (Sui et al., 2012), providing precise information regarding the location and timing of brain activity within the AON. Studies have used concurrent fNIRS and EEG during action execution (Zama et al., 2019), establishing the feasibility of such a study, but have not included an observation condition. Such work is important when considering potential therapeutic applications of AON findings: many have proposed motor imagery or action observation as rehabilitative techniques for those with motor impairments (Eaves et al., 2016; Caligiore et al., 2017), showing promising behavioral findings. AON studies using fNIRS can help pinpoint the neural changes afforded by these approaches, which could allow for the development of brain stimulation procedures for rehabilitative purposes. Conducting a study that incorporates both an execution and observation condition in an environment with greater external validity for action-observation processes than an EEG-fMRI study would afford is an important next step in studying the AON using fNIRS.

### Incorporate Wireless fNIRS Measurement to Further Optimize the External Validity of AON Research

New advances in NIRS systems allow wireless fNIRS measurement, a potentially valuable capability in studying the AON, particularly during action execution. The use of fNIRS has risen in recent years due to its affordability, portability, and relative tolerance to motion artifacts compared to other neuroimaging modalities. Subsequently, technology has progressed to further play to these assets by creating smaller, lighter systems that can be worn in a backpack for fully wireless studies. With the motor demand of AON paradigms, wireless fNIRS systems may be integral, particularly if the actions of interest involve more complex motion (e.g., walking). Wireless fNIRS devices have several advantages over wired fNIRS devices and fMRI. Not only are they less stressful for subjects due to freedom of movement and reduced sensitivity to motion artifacts (Pinti et al., 2020), they also offer greater external validity even compared to wired fNIRS systems. A wireless system allows actions to be assessed as a participant moves freely instead of eliciting actions through contrived experimental procedures, and observation of action to occur during normal social interactions with peers. Well-designed experiments that incorporate activities involving live, naturalistic social interaction between agents (e.g., games requiring turn-taking) could contribute to a better understating of the AON, particularly in the context of complex social interactions and its relationship with higher cognitive functions. Recent advances in fNIRS hyperscanning (conducting measurements on two people at once) also provide the opportunity to examine inter-brain activation (Czeszumski et al., 2020). The method could be particularly valuable in investigating the AON, as it has shown promise in similar paradigms, such as imitation tasks (Holper et al., 2012), and would allow questions regarding whether neural synchrony between social partners relates to AON function to be further assessed. The AON has been associated with social cognition, namely empathy (Gutsell and Inzlicht, 2010; Perry et al., 2010), the theory of mind (Pineda and Hecht, 2009), biological motion (Ulloa and Pineda, 2007), and language (Théoret and Pascual-Leone, 2002; Tamura et al., 2012; Jenson et al., 2014), but these links are still weak and controversial, and wireless fNIRS and hyperscanning protocols could help elucidate these relationships.

### Adapt fNIRS AON Paradigms for Use in Infants and Young Children

Another crucial application for fNIRS in investigating the AON is with developmental populations, such as infants, toddlers, or children. In comparison with fMRI, which requires immobility from infant/child participants, fNIRS has a relative tolerance for infant/child motion (Lloyd-Fox et al., 2010) and allows relatively shorter scanning times. Also, fNIRS is silent and allows participants to sit upright, as opposed to laying in a scanner bore, making it a more child-friendly imaging environment. Due to its temporal resolution (order of seconds) fNIRS is particularly convenient for paradigms that include live stimuli or social interaction (Matsui et al., 2014; Hakuno et al., 2018; Bhat et al., 2019). Most AON studies using fMRI use video or other visual stimuli as this is all that can be presented to a participant in the scanner (Filimon et al., 2007; Caspers et al., 2010; Molenberghs et al., 2012; Savaki and Raos, 2019), but fNIRS permits both participant and experimenter to reach for objects in real-time, allowing for a more ecologically valid paradigm. Further, fNIRS is less prone to motion artifact compared to EEG, but still comfortable and easy to set up in developmental populations. Many additional factors make fNIRS a modality well suited for infants and young children. For one, their skull is thinner (Beauchamp et al., 2011), allowing for propagation of light into the brain and more sensitive measurement than in adults. Also, infants and children have less hair, which aids in establishing a better fNIRS signal as hair results in the impaired optical coupling between probe and scalp (McDonald and Perdue, 2018). As mentioned before, one limitation of fNIRS compared to fMRI is an inability to map onto specific brain structures and localization of brain activity is based on external fiducials and position of optodes in the scalp. However, fNIRS-fMRI co-registration studies have shown that fNIRS channels can reliably be registered in the frontal and temporal cortex (Lloyd-Fox et al., 2014; Matsui et al., 2014), and a number of studies have shown that the 10-10 system serves as a good frame of reference in infants (Tsuzuki et al., 2017). In light of recent advances in creating infant and pediatric brain atlases from resting state MRI data (Zhang et al., 2019), we’ve gained the ability to localize cortical regions through proper measurement of the scalp and probe position and registration to a developmentally appropriate atlas, improving this limitation in the context of pediatric fNIRS studies. For all the reasons stated in this review article, fNIRS provides a promising tool to study the AON, particularly to understand its developmental trajectory. Attentional, motor, and social skills undergo tremendous growth during the first year of life, and by tracking their neural correlates in a more naturalistic environment through fNIRS, we can not only identify regions of the AON but also integrate these findings with behavioral measures of infant social behavior. This would provide a comprehensive understanding of whether AON functioning is related to social brain development, as indicated by various theories that have implicated the AON or “mirror neuron” system.

### Investigating the AON in Populations With Developmental Delays Using fNIRS

Due to the aforementioned theories implicating AON function in social development, fNIRS can provide a unique opportunity to study the AON in disorders affected by deficits in social development, such as neurodevelopmental disorders (e.g., autism spectrum disorder). While ASD is well characterized behaviorally, the underlying neurocognitive mechanisms of the disorder are still not understood. Oberman et al.’s (2005) seminal article on the link between ASD and the mirror neuron network led the scientific community to further examine the relationship between the AON/mirror neuron network and the ability to understand and imitate other’s behaviors in ASD populations. The debate is still ongoing (Hobson and Bishop, 2017) partially due to study design limitations (non-naturalistic/cross-sectional stimuli) and brain measurements used (mostly EEG). Regarding the former, fNIRS has successfully been used to examine differences in brain function associated with ASD risk (Fox et al., 2013; Lloyd-Fox et al., 2013), using social-emotional paradigms that include pre-recorded visual and auditory stimuli. However, fNIRS can also be used during naturalistic stimuli, like social interaction/AON paradigms, which could capture neural differences associated with ASD that might only be evident in live interactive interactions (Rolison et al., 2015; McDonald and Perdue, 2018). Another important application of fNIRS when researching neurodevelopmental disorders/atypical brain development is in the use of longitudinal designs. Most fNIRS studies aimed at examining the early neural development of ASD use cross-sectional designs in already diagnosed children, or in a group of infants with known risk factors such as an older sibling with the disorder (high-risk sibling); these designs don’t allow for the assessment of whether neural differences between groups are specific to diagnosis or are just associated with a familial risk of ASD (McDonald and Perdue, 2018). By tracking brain activity in response to an action-observation paradigm across multiple time points, researchers can better understand the developmental trajectory of the AON by answering questions such as what areas of the brain are activated, does activation change with development, or is the AON activated differently depending on the behavioral characteristics of the sample? This research is worth pursuing as there is a possibility that the AON could provide for a predictive biomarker a later diagnosis or be used as an intervention outcome measure.

## Conclusion

The current review article focused on the suitability of fNIRS in the study of the AON, emphasizing the potential of this neuroimaging technique in the context of the study of action-observation and action-execution. Although it has been over two decades since the AON (also referred to as mirror neuron network) was first described in humans (Iacoboni et al., 1999) there is still an ongoing debate in the scientific community regarding its neural correlates and functional importance. This is in part due to the limitations of modalities historically used to study the AON, namely EEG and fMRI. The former lacks the spatial resolution needed to localize the source of the signal and presents issues with potential confounds from other sources of brain activity, namely attention; the latter lacks ecological validity and does not allow the person to engage in a proper range of action-execution tasks. In this article, we present arguments showing why fNIRS is a valuable tool for filling this gap in the literature and can help answer some of the questions that remain in the AON field.

## Author Contributions

The manuscript was conceived by EC and HM. The literature search, article selection, and article review were conducted by EC, HM, JM, DH, and KK. The manuscript was drafted and edited by EC, HM, JM, DH, KK, NF, and AG. All authors contributed to the article and approved the submitted version.

## Conflict of Interest

The authors declare that the research was conducted in the absence of any commercial or financial relationships that could be construed as a potential conflict of interest.
