# Supplementary material for: Characterizing the Action-Observation Network Through Functional Near-Infrared Spectroscopy: A Review
Source: Front Hum Neurosci. 2021 Feb 18;15:627983. doi: 10.3389/fnhum.2021.627983 (PMC7930074; doi:10.3389/fnhum.2021.627983)
Supplement: Supplementary file 1 [file Table_1.docx]

Table 1

*Summary of the 14 articles fitting the search criteria for the present review.*

| **Paper** | **Sample size(s)** | **Handedness** | **Sample age**  **Mean (SD)**  ***range*** | **Task description** | **Paradigm conditions** | **Presentation style of observation** | **Number of trials per condition** | **fNIRS system** | **# of channels** | **Probe position** | **Covered ROIs** | **Summary of findings**  **(AON indications in BOLD)** |
| --- | --- | --- | --- | --- | --- | --- | --- | --- | --- | --- | --- | --- |
| Kajiume et al. (2013) | TD = 6; PDD = 6 | Right | PDD: 10.7 (2.9)  *range: 8-14* TD: 10.9 (1.6)  *range: 9-13* | Bottle opening & closing | Imitate, observe | Video | 4 | Hitachi ETG-100 | 24 | Bilateral | Bilateral temporal lobe (T3/T4) | Comparisons focused on differences between diagnostic groups.  PDD showed lower activation than TD, specifically the PDD groups showed less activity during OBS in the right hemisphere. |
| Sun et al. (2018) | 30 | Right | 23 *range: 18-27* | Table Setting Paradigm | Execute, observe | Live with AV prompt | 4 | Shimadzu LABNIRS | 54 | Unilateral (L) | L PMC, L IFG, L SPL, L rostral IPL | **Activation during EXE and OBS trials in L PMC, L SPL, and L IFG.** L rostral IPL active in EXE, but not OBS. EXE trials produced greater activation than OBS trials. |
| Balconi & Cortesi (2015) | 18 | Right | 31 (6.7) | Transitive & intransitive gestures | Execute, observe | Video | 48 | NIRx | 24 | Unilateral (L) | L PMC, L SM1, L PPC | **Activation during EXE and OBS trials similar in L PPC.** **Activation was seen in L SM1 and L PMC during both EXE & OBS compared to rest;** however, activation was higher in L PMC during EXE than OBS. EXE showed greater activation than OBS in PMS and SM1 |
| Balconi, Crivelli, & Cortesi (2017) | 13 | Right | 28 (5.9) | Transitive & intransitive gestures | execute and observe | Video | 48 | NIRx | 24 | Unilateral (L) | L PMC, L SM1, L PPC | L PMC & L SM1 showed greater activation during EXE than OBS.  **L PPC showed similar activation in OBS and EXE.**  Generally, EXE trials produced greater activation than OBS trials. |
| Bhat et al. (2017) | 15 | Right | 22.6 (0.7)  *range: 19-27* | Table Cleaning Paradigm | Execute, Observe, Synchronous | Live | 8 | Hitachi ETG-4000 | 24 | Bilateral | L & R SA (supero-anterior region; IFG/SM1) L & R IP (infero-posterior region; angular/supramarginal gyri & IPL) L & R IA (infero-anterior region; superior/middle temporal gyri & STS) | Bilateral SA & contralateral IP active during EXE.  **Bilateral IA active in both EXE & OBS.**  EXE > OBS across ROIs. |
| Crivelli, Rueda, & Balconi (2018) | 20 | Right | 24.15 (1.59) *range: 22-28* | Transitive gesture | Execute, observe, listen, execute-listen, observe-listen | Video | 22 | NIRx NIRScout | 16 | Bilateral | L & R ventral PMC, L & R somatosensory | EXE > OBS across ROIs Activity in R hemisphere > L for OBS; whereas activity in L > R for EXE. |
| Egetemeir et al. (2011) | 17 | Right | 23.5 (3.3) | Table Setting Paradigm | execute-joint action, execute-simultaneous, execute-solo action (SA), observe | Live | 8 | Hitachi ETG-4000 | 52 | Unilateral (L) | L IFG, L IPL | **L IPL active in both EXE (SA) and OBS.** |
| Koehler et al. (2012) | 39 | Right | 21.8 *range: 19-30* | Table Setting Paradigm | execute, observe-allocentric, observe-egocentric | Video | 8 | Hitachi ETG-4000 | 52 | Unilateral (L) | L frontal, L motor, L parietal | L frontal gyrus, SM1, and occipital lobe active during EXE. L STS active in OBS. **L IPL active in both EXE and OBS.** |
| Holper et al. (2010) | 15 unilateral 8 bilateral | Right | 26,  *22-33* | VR observation, imagery and imitation | Observation, observation+motor imagery, motor imagery, and imitation | Video; virtual reality environment. | 10 | Custom | 8 | Unilateral (L) for 15 subjects; bilateral for 8 subjects. | F3 & F4 | Unilateral group: Imitation showed activation in L hemisphere, OBS did not. Bilateral group: Imitation and OBS both showed activation in both R hemisphere however only Imitation showed activation in L hemisphere. |
| Shimada & Abe (2010) | Experimental group=12  Control1=12  Control2=8 | Right | Exp: 22.5 (1.3); Control1: 23.3 (1.0); Control2: 22.9 (1.0) | Rock Paper Scissors | Observation, Execution-instructed, Execution-autonomous, Static | Video | 12 | Shimadzu OMM-3000 | 10 | Unilateral (L) | L SM1 | Most channels over L SM1 active during EXE. **One channel over L superior SM1 was active in OBS and EXE.** |
| Balconi et al. (2015) | 13 | Right | 28 (5.9) | Transitive & intransitive gestures | Execute, observe | Video | 48 | NIRx | 24 | Unilateral (L) | L PMC, L SM1, L PPC | **PPC activation during EXE = OBS.** PMC & SM1 activation during EXE > OBS |
| Shimada & Abe (2009) | 13 | Right | 22.1 (0.60)  range*: 21–23* | Rock Paper Scissors | Execute, observe (with a variety of conditions) | Video | 54 observe; 8 execute | Shimadzu OMM-3000 | 10 | Unilateral (L) | L SM1 | L SM1 was active in both OBS and EXE conditions; no contrasts between the conditions were performed. |
| Xu et al. (2019) | 23 | Right | 20.7 (1.6)  *range: 18–25* | Table Setting Paradigm | Execute, observe; before and after exercise | Live | 8 | NIRx NIRSport | 14 | Unilateral (L) | L IFG, L PMC, L rostral IPL, and L SPL | All ROIs were active in both EXE and OBS conditions prior to exercise manipulation. |
| Zhang et al. (2019) | Athletes=4, controls=20 | Right | Athletes=23.75CG=22.65 | Walking | Execute, observe, motor imagery | Video | 1 | Shimadzu LABNIRS | 45 | Bilateral | Pars triangularis, SMA, MFG, dlPFC, SM1 | ROIs that differed based on condition included: IFG, dlPFC, PMC, SMA, & SM1; however, follow-up contrasts are not reported. |
| **Notes:** TD=Typically developing; PDD=Pervasive developmental disorder; PMC=premotor cortex, SM1=primary sensory motor cortex; PPC=posterior parietal cortex; SMA=supplementary motor area; IFG=inferior frontal gyrus; IPL=inferior parietal lobule; SPL=superior parietal lobule; STS=superior temporal sulcus; dlPFC=dorsolateral prefrontal cortex; MFG=middle frontal gyrus | | | | | | | | | | | | |
